# A *Phytophthora infestans CRN1-*derived small RNA is predicted to target the potato immune regulator *EDS1*

**DOI:** 10.3389/fpls.2026.1791978

**Published:** 2026-04-10

**Authors:** Shailja Singh, Xinyi Hu, Christina Dixelius

**Affiliations:** Department of Plant Biology, Swedish University of Agricultural Sciences, Uppsala, BioCenter, Uppsala, Sweden

**Keywords:** Argonaute, CRN effectors, EDS1, extracellular vesicles, Phytophthora infestans, potato, small RNAs

## Abstract

The late blight pathogen, *Phytophthora infestans* (*Pi*), causes severe damage to plants in the Solanaceae family. Although knowledge regarding the *P. infestans-*mediated manipulation of critical components in the plant defense system is growing, many questions remain unanswered. Herein, we aimed to examine the role of Argonaute 1 (AGO1) associated small RNAs in this interaction. Of particular interest was the early communication between the host and the pathogen. To visualize the cellular dynamics underlying potential cross-kingdom RNA trafficking, we first examined the localization and accumulation patterns of plant extracellular vesicles (EVs) and multivesicular bodies (MVBs) using a handful of markers. MVBs were present not only at the plant plasma membrane but also in the germ tube of the invading pathogen. The enrichment of MVBs decreased as the infection process proceeded. At 3.0 days post-inoculation, co-localization between AGO1 from *P. infestans* and *St*ARA6 was not seen even at the swollen tip of the germ tube. Three Crinkler effector genes encoding small RNAs were found after coimmunoprecipitation, sequencing and extensive bioinformatic analysis. *PiCRN1* caused more severe disease compared with *PiCRN3*, which carries a typical Crinkler (CRN) LFLAK domain. This difference may result from activation of a *CRN1*-derived siRNA predicted to target the enhanced disease susceptibility 1 (*EDS1*) gene in potato. To examine whether the observed phenotypic effects can be attributed to any EV cargo from the potato host, we set up a procedure to isolate EVs from *P. infestans*-infected potato leaves. However, the tiny EV yield obtained during the early infection phase prevented us from such analysis. The understanding of effector cell trafficking and small RNA reprogramming of host plant genes remain elusive in this pathosystem.

## Introduction

The membrane-trafficking system, first discovered in mammalian cells, has subsequently been found in most eukaryotes and bacterial species (Couch et al., 2021; Wang et al., 2023). Cellular communication is based on extracellular vesicles (EVs), a heterogeneous group of organelles with different shapes and sizes, enclosed by lipid bilayer membranes that envelope a wide range of cargo considered as waste products to be transported out of the cell (exocytosis) or molecules targeted to other organelles (Stotz et al., 2022). These vesicles also mediate transport in the opposite direction (endocytosis), which includes the cellular import of a range of external molecules, including those from intruders that attempt to invade the plant. All cells can secrete various types of membrane-enclosed vesicles, which are involved in multiple physiological and immune-related processes essential for cell survival (Van Niel and Raposo, 2018). In brief, intracellular trafficking involves steps such as vesicle assembly and processing in the endoplasmic reticulum and Golgi apparatus, followed by their transport to the multivesicular body (MVB) and lysosome/vacuole, with a destination at the plasma membrane (Schlacht et al., 2014; More et al., 2020; Singh et al., 2023). The transport processes in the cell are mediated by the cytoskeleton network, where the so-called motor proteins, together with regulatory proteins and small GTPases, master vesicle movements along the filaments (Chebli et al., 2021; Yuan et al., 2023). The “Ras-like protein from rat brain” or RAB GTPases are the main components in this process (Zerial and McBride., 2001). The corresponding RAB GTPases in *Arabidopsis* are the ARA proteins, where ARA6 is a commonly used marker for MVB biogenesis (Ueda et al., 2001; Li et al., 2018). MVBs are the central components of endosomal trafficking and EV biogenesis (Cui et al., 2020) and may represent important sites where membrane transport pathways in the host intersect with pathogen invasion (Rutter and Innes, 2017; Cai et al., 2018). Tetraspanins (TETs) are another group of conserved transmembrane proteins in eukaryotic cells, with 17 genes identified in *Arabidopsis* (Qin et al., 2024). TET8 and TET9 play important roles in the secretion of plant EVs that mediate the trafficking of host small RNAs into fungal pathogens to silence their virulence genes (Cai et al., 2018). The endocytic and endosomal trafficking systems in plants have garnered increasing interest, as it has become evident that both pathogens and plant host molecules can utilize this membrane-based secretion system to invade organisms with whom they are in close contact.

The late blight disease, caused by the oomycete pathogen *Phytophthora infestans*, remains a major problem, particularly for potato and tomato crops, worldwide (Nowicki et al., 2012). This pathogen is heterothallic, with the capacity to produce a large number of spores (Fry, 2008), and its genome is dense, containing numerous potential effector candidates (Haas et al., 2009). Since 1876, when de Bary (1876) first presented his study on this pathogen, different stages of host plant infection by the pathogen have been studied. However, several fundamental questions, including when and what types of effectors are delivered during the initiation and progress of the late blight disease in the potato tissue, have not been fully answered. In brief, the infection starts with germinating spores that land on a plant host and form an appressorium-like swelling at the end of the germ tube (Whisson et al., 2016; Boevink et al., 2020). Small appressoria are formed, and enzymes are secreted to degrade the plant cuticle and cell walls. After they enter the plant cell, intercellular hyphae start growing from the infection vesicle, ramify the tissue, and form special structures called haustoria. The specific tight physical connection observed between haustoria, and the plant endomembrane system suggests that haustoria play a central role in the exchange of molecules between the two organisms with different effectors (Wang et al., 2018). The *P. infestans* genome harbors more than 500 predicted RXLR effectors, named after the conserved amino acid motif, which follows the signal peptide in proteins (Haas et al., 2009; Pais et al., 2018). However, the RXLR effectors represent only a small share of the wide range of molecules secreted by this pathogen (Dagdas et al., 2018). Crinkler (CRN) effectors constitute an additional large group of 196 predicted genes and 255 pseudogenes (Haas et al., 2009). The spores of *P. infestans* encyst on the plant surface, followed by their germination, germ tube growth, and host tissue penetration. Subsequently, *P. infestans* forms haustoria in the invaded host cells during the early biotrophic phase of the infection. Studies on secreted effectors from *P. infestans* have often been centered on this specialized structure (Wang et al., 2017; Boevink et al., 2020).

In this study, we aimed to investigate potential cross-kingdom events that occur early in the plant–*P. infestans* interaction, before haustoria formation. We found plant EVs in the germ tubes of *P. infestans* before their entry into the host. Data from *Pi*AGO1 co-precipitation and small RNA sequencing provided information on siRNAs from CRN effectors. Of particular interest was CRN1, which lacks a typical LFLAK motif but promoted infection in *Nicotiana benthamiana*. This function could most likely be explained based on a *CRN1*-derived siRNA that is predicted to target the enhanced disease susceptibility 1 (*EDS1*) gene, which codes for the EDS1 protein, a central component in the immune system.

## Materials and methods

### Plasmids

The potato (*Solanum tuberosum*, *St*) orthologs of the genes coding for *Arabidopsis* Rab/Ypt GTPase ARA6 (Ueda et al., 2001) and TET8 (Boavida et al., 2013) were amplified from the leaves of the cultivar Desirée and inserted in the Gateway plasmids pGWB561 downstream of the 35S promoter or in pGWB555 with the same promoter and carrying a red fluorescent protein (RFP) tag. The primers used in the cloning experiments are listed in **Supplementary Table 1**. A plasmid harboring *StAGO1*a with a DNA fragment encoding the green fluorescent protein (GFP) tag was also included in the study (Persson Hodén et al., 2021). The predicted *CRN* genes, i.e., *CRN1*, *CRN2*, and *CRN3*, were cloned from the *P. infestans* strain 11388 and inserted into the Gateway plasmid pGWB506 with a GFP tag.

### Plant materials and pathogen strains

*Nicotiana benthamiana* and potato cultivar ‘Desirée’ were cultivated as described previously (Li, 2011; Jahan et al., 2015). Two *P. infestans* strains were used, i.e., 11388 and 88069. The culture conditions and methodology used for inoculating *P. infestans* were as outlined earlier (Vetukuri et al., 2011; Jahan et al., 2015).

### Agroinfection and confocal microscopy

For all analyses, we used 6.0-week-old *N. benthamiana* wild-type plants (Hu et al., 2022). Leaves were agroinfiltrated with pGWB506 containing *35S::PiAGO1-GFP*, *35S::StAGO1a-GFP* or pGWB561 harboring *35S::StTET8-RFP*, *35S::StARA6-RFP*, *35S::PiCRN1-GFP*, *35S::PiCRN2-GFP*, and *35S::PiCRN3-GFP.* The same leaves were inoculated with the *P. infestans* strain 11388, 1.0-day post-agroinfiltration. Responses were monitored daily and recorded using an LSM800/LSM780 NLO confocal microscope (Zeiss, Oberkochen, Germany). The excitation/detection wavelengths for GFP and RFP were 488/499–547 nm and 488/532–588 nm, respectively. The confocal analysis on the different materials was repeated at least four times.

### Quantitative reverse transcription–polymerase chain reaction

Total RNA was isolated from potato leaves, and EVs were collected using the RNeasy Plant Mini Kit (Qiagen, Sollentuna, Sweden) and TRIzol reagent (Thermo Fisher Scientific, Waltham, MA, USA). cDNA synthesis was performed using the Maxima First Strand cDNA Synthesis Kit (Thermo Fisher Scientific, Waltham, MA, USA). The analysis was performed on three independent biological replicates, with 30 leaves per sample, using the Maxima SYBR/Fluorescein qPCR Master Mix (Thermo Fisher Scientific, USA). For pairwise comparisons, a two-tailed Student’s *t*-test was used. Statistical significance thresholds were set at *P* < 0.05 (*), *P* < 0.01 (**), and *P* < 0.001 (***). Replicate numbers and statistical details are provided in the figure legends.

### Isolation of plant extracellular vesicles

Potato EVs were isolated from apoplastic washing fluid. The isolation procedure used was based on a combination of previously described methods (Rutter and Innes 2017; Huang et al., 2021). In brief, 6.0-week-old plants were inoculated with the *P. infestans* strains 88069 or 11388 (Åsman et al., 2016; Vetukuri et al., 2011). Sixty uninfected (control) and infected leaves were collected 5.0 days post-inoculation (dpi). Their surfaces were carefully cleaned using sterile water. Apoplastic washing fluid was collected after three centrifugation steps. First, the collected wash samples were centrifuged at a speed of 900 × *g* to remove cell debris, followed by ultracentrifugation at 40, 000 × *g* and 100, 000 × *g* to obtain the P40 and P100 fractions, respectively. The EV pellet was resuspended in 1 x PBS for further analysis. Samples with potential EVs were first monitored using transmission electron microscopy (TEM). To further assist in the evaluation, the samples were stained with DiOC_6_ (3, 3′-dihexyloxacarbocyanine iodide; Otsuga et al., 1998), a lipophilic dye that selectively labels the endoplasmic reticulum, vesicle membranes, and other membrane-bound organelles (Rutter and Innes, 2017) and checked for fluorescent signals. The isolated EVs were prepared for the qRT–PCR analysis. Infection using *P. infestans* strains was performed as described earlier (Persson Hodén et al., 2021).

### TEM analysis

The TEM analysis of potato EVs was performed following Corona et al. (2023). For negative staining, a 5.0 µl drop of the EV fraction (P100) was carefully placed onto a carbon-coated copper mesh grid (TED PELLA INC., (Caspilor AB Lidingö, Sweden), followed by gentle washes with sterile water. A 2.0% uranyl acetate solution was used for staining. Air-dried grids were examined under a Tecnai G2 Spirit BioTwin 80 kV transmission microscope from FEI (Hilsboro, USA) at the Biological Visualization (BioVis) platform, Uppsala University, Sweden.

### Bioinformatics and statistical analyses

The datasets for *StAGO1a* sequences were derived from a small RNA pull-down assay of infected and uninfected plants. Libraries were prepared using the Illumina TruSeq Small RNA Kit and sequenced on an Illumina NextSeq 2000 platform at SciLifeLab (Solna, Sweden). In parallel, data from degradome analysis, and small RNA datasets from *P. infestans* combined with information on effectors, tasiRNA loci and potato resistance genes were all integrated and run in a convolutional neural network using a smartPARE R package. Details on materials and analysis can be found in Persson Hodén et al., 2021. The sequence data can be found at the NCBI Gene Expression Omnibus (GEO; https://www.ncbi.nlm.nih.gov/geo/) under accession number GSE163382.

## Results

To visualize the cellular dynamics underlying potential cross-kingdom RNA trafficking, we first examined the localization and accumulation patterns of plant EVs and MVBs at different cellular levels during the infection of *N. benthamiana* leaves by *P. infestans*. Based on the enrichment of the *St*ARA6 and *St*TET8 markers, we inferred that both MVBs and EVs increased upon *P. infestans* infection compared with those in the uninfected control conditions (**Supplementary Figures 1A–D**). This activation of the endosomal trafficking and secretion machinery in response to pathogen attack was expected, as similar observations have been reported from the *Arabidopsis*–*Pseudomonas Pst* DC3000 system, followed by other examples (Wang et al., 2014; Yuen et al., 2023).

**Figure 1:**
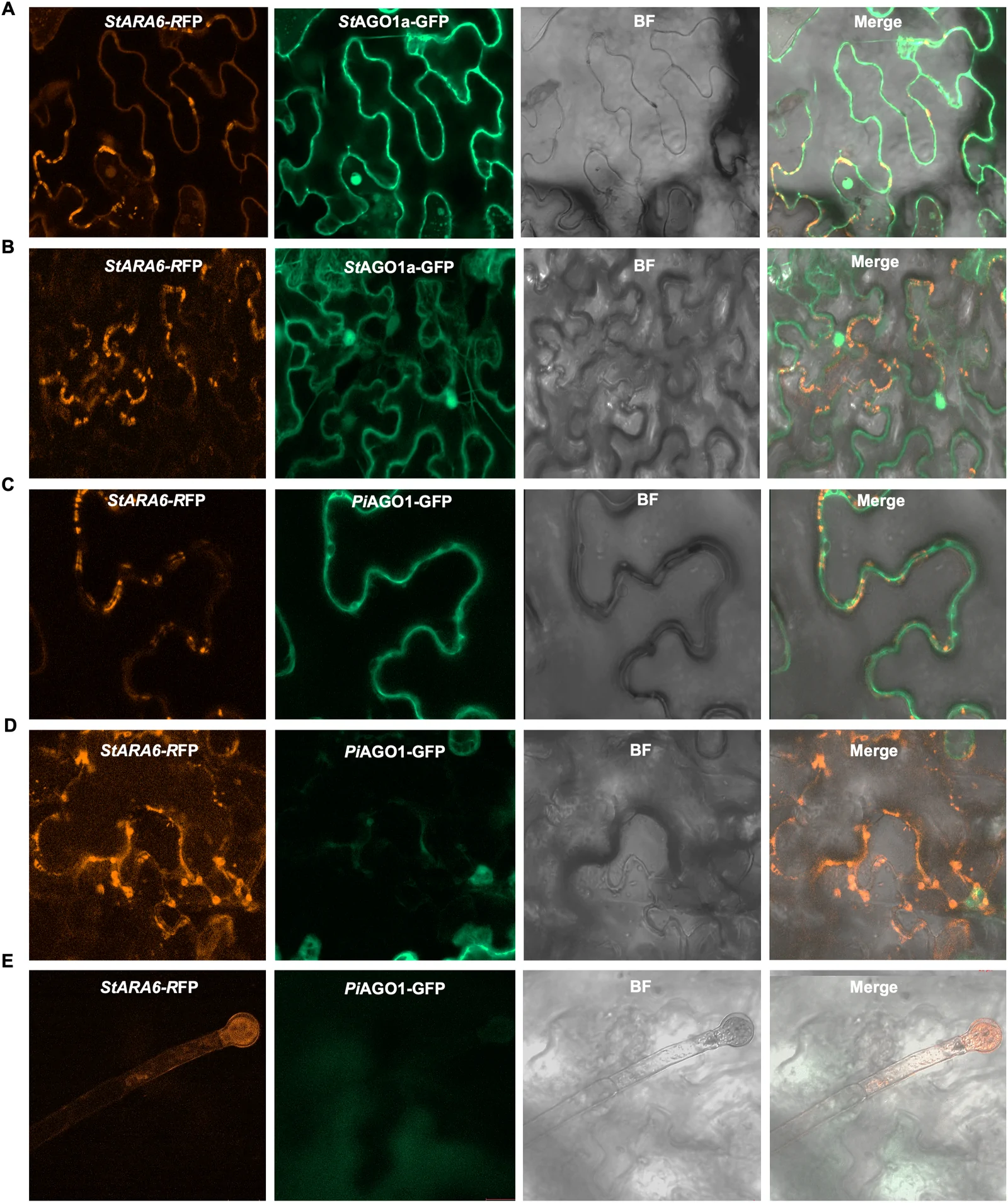
Co-localization of extracellular vesicles with Argonautes (AGOs) in *Nicotiana benthamiana* with and without *Phytophthora infestans* infection. **(A)***St*ARA6-RFP marker co-localized with *St*AGO1a-GFP, without infection, **(B)***St*ARA6-RFP marker co-localized with *St*AGO1a-GFP upon *P. infestans* infection. **(C)***St*ARA6-RFP and *Pi*AGO1-GFP markers without infection, **(D)***St*ARA6-RFP and *Pi*AGO1-GFP markers upon *P. infestans* infection. **(E)***St*ARA6-RFP and *Pi*AGO1-GFP markers upon *P. infestans* infection. The confocal images are here focused on the growth of the mycelia of *P. infestans* on the leaf surface. Scale bars represent 10 μm. White arrows indicate the localization of extracellular vesicles (EVs). Confocal images are representative of at least four independent experiments.

**Figure 2:**
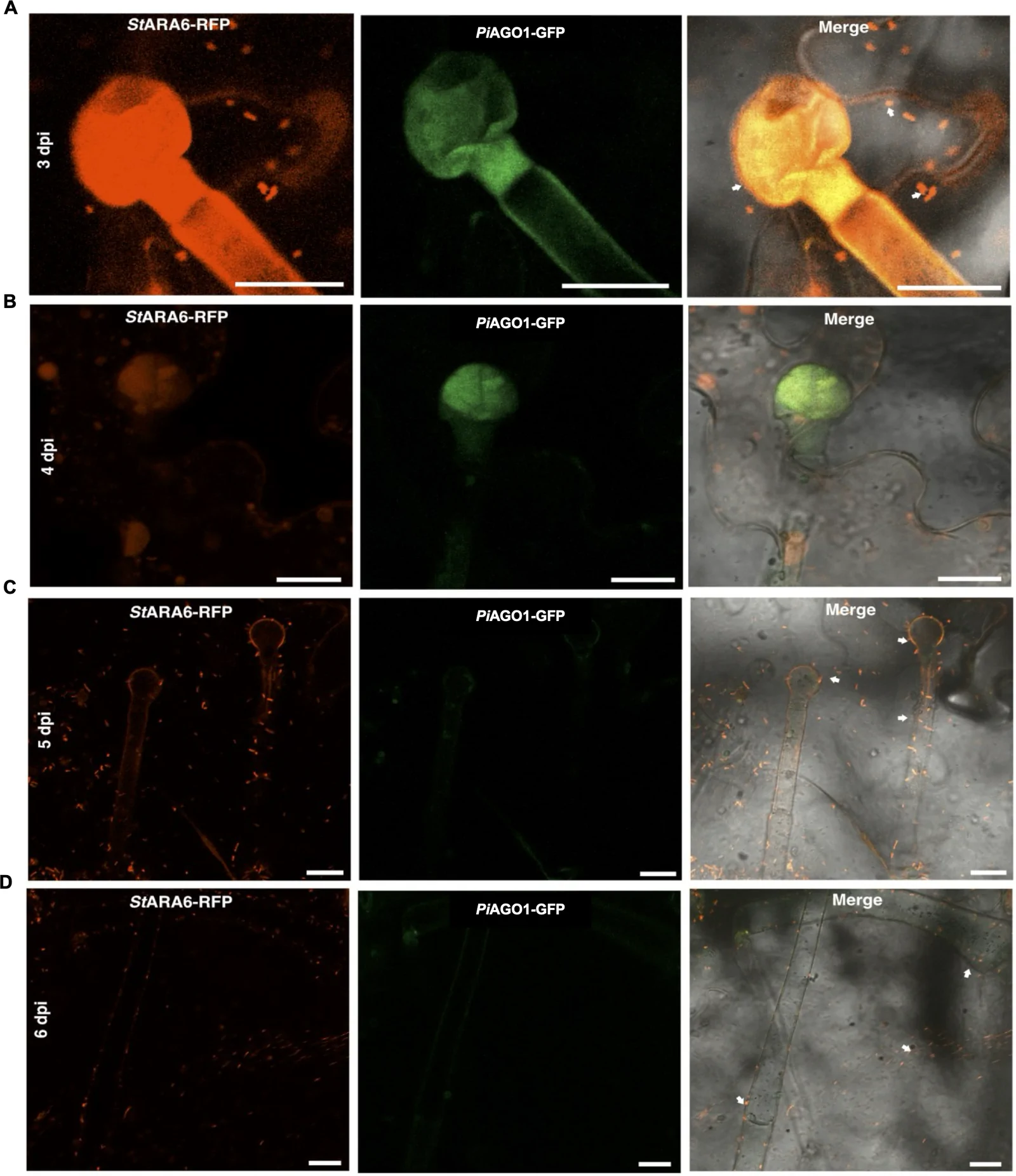
Co-localization of *St*ARA6-RFP marker with *Pi*Ago1-GFP during *Phytophthora infestans* infection in *Nicotiana benthamiana* over a 3–6 days post-infection (dpi) time course shown in **(A)** to **(D)**. Scale bar represents 20 μm. The white arrows indicate the localization of extracellular vesicles (EVs). Confocal images shown are representative of at least four independent experiments.

**Figure 3:**
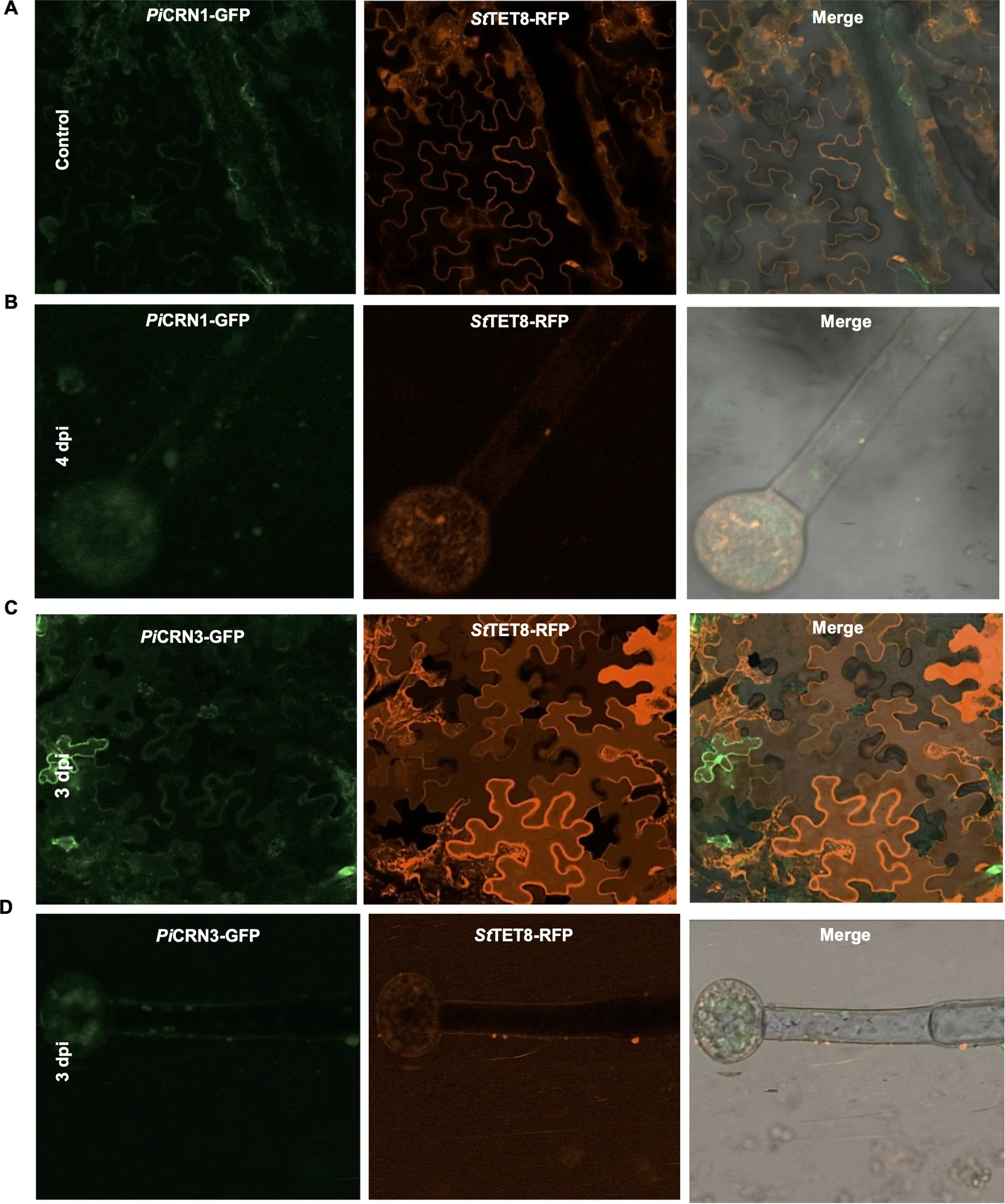
Confocal microscopy of the transiently expressed potato extracellular vesicle (EV) marker *St*TET8-RFP and green fluorescent protein (GFP)-tagged *Pi*CRN proteins in *Nicotiana benthamiana*. **(A)** No infection. Scale bar represents 50 μm. **(B–D)** Images showing **(B)***Pi*CRN1-GFP at 4.0 days post-infection (dpi), **(C)***Pi*CRN2-GFP at 3.0 dpi, **(D)***Pi*CRN3-GFP at 3.0 dpi with *Phytophthora infestans*. Scale bars represent 10 μm. Panels A and C show low-magnification overviews, while **(B)** and **(D)** show higher-magnification views of the host-pathogen interface. Images are representative of at least four independent experiments. *Pi*CRN1-GFP images were captured at 4.0 dpi to better visualize localization at the host–pathogen interface.

Next, we examined the cellular localization of *St*AGO1a after agroinfiltration. We found that *St*AGO1a-GFP co-localized with the *St*ARA6-RFP marker (**Figures 1A–D**). MVBs were seen not only at the plant plasma membrane but also in the germ tube of the invading pathogen (**Figure 1E**). The latter prompted us to look closely at events that occur before 5.0 dpi. At 3.0 dpi, the co-localization between *Pi*AGO1 and *St*ARA6 was not seen even at the swollen tip of the germ tube (**Figure 2A**). The enrichment of MVBs decreased as the infection process proceeded (**Figures 2B–D**). Based on the *St*AGO1a pull-down and sequencing data (Persson Hodén et al., 2021), we only identified siRNAs originating from *P. infestans* that are predicted to target host genes in potato. No potato siRNAs predicted to target *P. infestans* genes were detected (**Supplementary Tables S2**, **S3**). Notably, three CRN effectors were found among the listed candidates for the *P. infestans* siRNA source. The three *CRN* genes were individually cloned and tagged with GFP. The *P. infestans* strains were combined with the *St*TET8-RFP marker in assays using *N. benthamiana.* The enrichment of potato EVs after the expression of *CRN1* and *CRN2*, compared with that after the expression of *CRN3*, was noticed (**Figure 3**).

**Figure 4:**
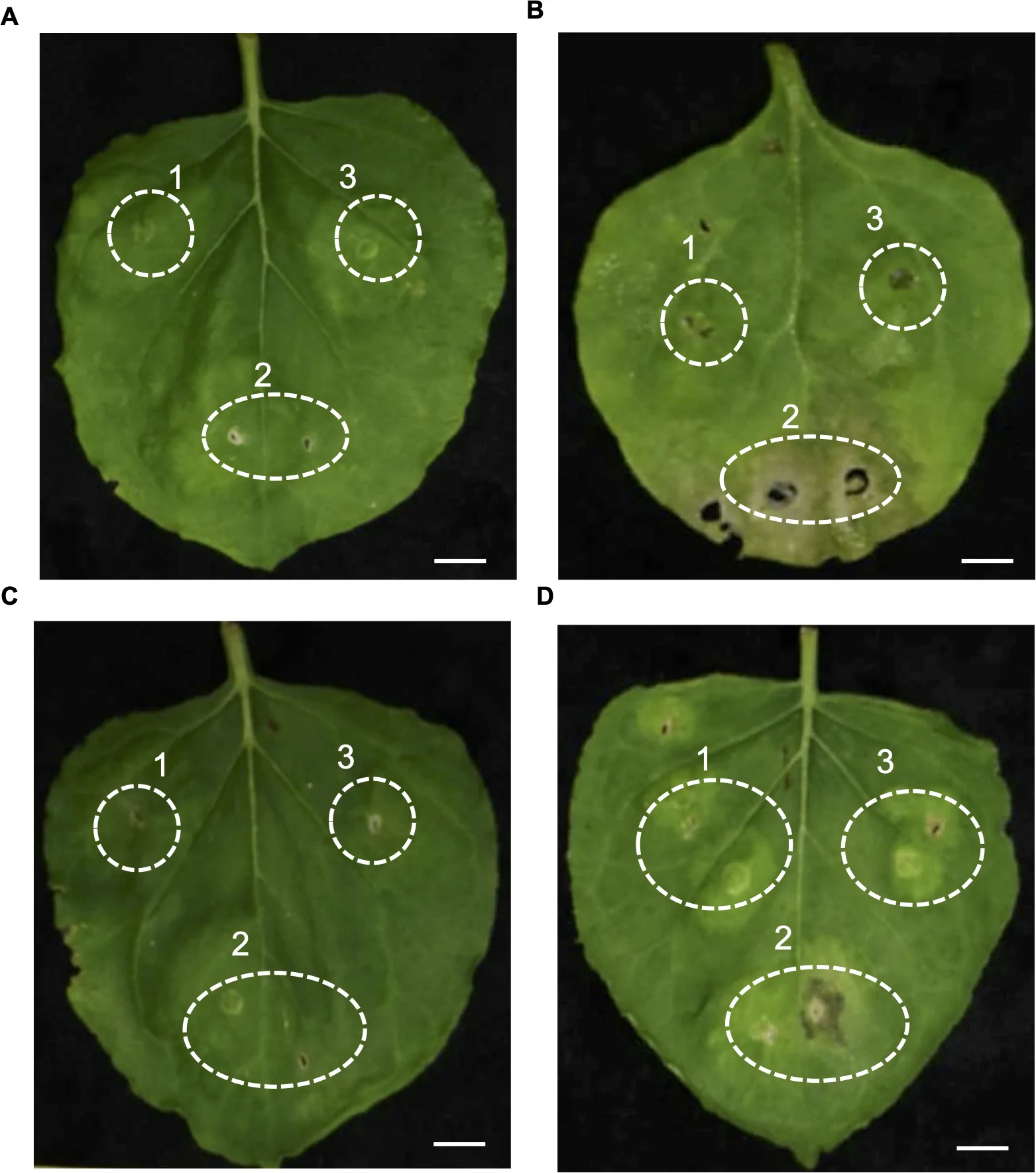
*PiCRN1* compromises the immune response compared to *PiCRN3*. **(A)** 1. Empty vector, 2. *Pi*CRN1 plasmid, and 3) *Pi*CRN1 and *St*TET8. No infection. **(B)** 1. Empty vector, 2. *Pi*CRN1 plasmid, and 3) *Pi*CRN1 and *St*TET8. *Phytophthora infestans* infection. **(C)** 1. Empty vector, 2. *Pi*CRN3 plasmid, and 3) *Pi*CRN3 and *St*TET8. No infection. **(D)** 1. Empty vector, 2. *Pi*CRN3 plasmid, and 3) *Pi*CRN3 and *St*TET8. *Phytophthora infestans* infection. Images were captured at 5.0 days post-infection (dpi). Scale bars represent 1.0 cm.

Among the three CRN proteins, CRN3 is the only candidate that carries a typical LFLAK domain. We then checked whether cargo from plant EVs could potentially impact pathogen virulence by infecting *N. benthamiana* leaves with a wild-type *P. infestans* strain with plasmids carrying either *PiCRN1* or *PiCRN3* (**Figure 4**). Two observations were made: *PiCRN1*, promoted infection when applied externally, and *StTET8* (potato EVs) suppressed the extent of the infected area (**Figures 4A, B**). Similar phenotypic responses were seen in the case of *PiCRN3* (**Figures 4C, D**). However, *PiCRN1* caused more severe disease compared with that caused by *PiCRN3* (**Figures 4B, D**). This difference may be associated with the presence of an siRNA identified within the *CRN1* sequence that is predicted to target the *EDS1* gene in potato (PGSC0003DMT400083030) (**Supplementary Table 3**), suggesting a potential cross-kingdom small RNA-mediated interactions. To further explore whether the observed phenotypic effects could involve EV-associated cargo from the potato host, we established a procedure to isolate EVs from *P. infestans*-infected potato leaves (**Supplementary Figure 2A**). The final pellet from layer P100 was analyzed for EV size using TEM. The diameters of the EVs varied between 36.6 nm and 46.8 nm (**Supplementary Figures 2B–D**). Subsequently, the pellet contents were subjected to DiOC_6_ staining using the fluorescent dye DiOC_6_ to visualize vesicle membranes (**Supplementary Figure 2E**). Immunogold labelling of canonical EV markers was attempted, but technical issues during the blocking step prevented clear signal detection. Next, presence of *Pi*-miR8788 (Hu et al., 2022) in the different EV fractions were analyzed. In addition to being present in the initial leaf samples, this miRNA was enriched in the P100 fraction when both the 11388 and 88069 strains were used, but particularly when strain 11388 was used for infection (**Supplementary Figures 2F, G**). Subsequently, *Pi*-siRNA 5′-AACTACTCCATGAATGTCTCC-3′ and *Pi*-siRNA 5′-TTGGAGAGATGGAAAGACGG-3′, predicted to target the 40S ribosomal protein S8 and *EDS1* gene, respectively, were analyzed (**Supplementary Figures 3A, B**). The latter was only found in the initial leaf samples, whereas *Pi*-siRNA 5′-AACTACTCCATGAATGTCTCC-3′ was enriched in the EV fractions. This result suggests that the targeting of *EDS1* by small RNAs may require sufficient time to consolidate correct membrane proteins or other helpers and avoid degradation, and successfully enter the host tissue, most likely through a separate secretion pathway.

## Discussion

In this study, we monitored host-derived EVs and MVBs during the infection of plant leaves by *P. infestans*. An enrichment of these small membrane-bound vesicles in the host tissue was seen at an early stage of *P. infestans* infection. Similar observations have been made for several other plant pathogens, such as the powdery mildew fungus and *Colletotrichum* species (Rubiato et al., 2022; Koch et al., 2025). The role of membrane-bound vesicles in pre- and post-invasive host defense responses has been suggested in multiple studies on syntaxins, which are members of the SYP12 clade (Rubiato et al., 2022). We expected the syntaxin-related genes in potato to be the targets of small RNAs from *P. infestans*; however, no such candidates were found in the *Pi*AGO1 or *St*AGO1a datasets (Hu et al., 2022; Persson Hodén et al., 2021). The miR8788 from *P. infestans*, which targets the potato α/β hydrolase-type gene (*StABH1*) located in the plasma membrane, has been suggested to participate in translocation processes during the infection process (Hu et al., 2022). This miRNA was also present in the isolated EVs in the present study. Numerous plant processes are affected by the large repertoires of *Phytophthora* effectors (Fabro, 2022). It is well-documented that *P. infestans* secretes cell wall-degrading and -modifying enzymes, as well as RXLR effectors that translocate from haustoria into plant cells, where they localize in several organelles (Wang et al., 2017, 2019). CRN effectors are proteins composed of a variety of domains in different orders (Haas et al., 2009). They are a widespread class of proteins not only in oomycetes, but they also are present in several kingdoms (Zhang et al., 2016), whereof several species do not form haustoria. Thus, the translocation process into plant cells may involve more than one single route (Amaro et al., 2017). Likewise, how CRNs enter host nuclei and interfere with DNA where they often are implicated in cell death processes (Torto et al., 2003). The finding that *Phytophthora sojae* CRN63 induces, whereas *Ps*CRN115 suppresses cell death (Liu et al., 2011), demonstrates the multifunctional capacity of this effector group. Besides endogenous *CRN* gene regulation in *P. parasitica* siRNAs (Jia et al., 2017) cross-kingdom events by *CRN* effectors encoding a siRNA that potentially targets a host gene implicated in plant defense has so far not been reported.

The finding that CRN1 lacks a signal peptide but can promote infection can most likely be attributed to the siRNA-mediated targeting of the *EDS1* gene, which impairs the activation of host resistance genes. Interestingly, while the selective enrichment of *Pi-miR8788* and 40S ribosomal protein siRNAs in EVs suggests a specialized trafficking system for certain small RNAs, the absence of the *EDS1*-targeting siRNA from these fractions indicates that *P. infestans* utilizes diverse delivery mechanisms. These data discriminate our observations from nonspecific co-pelleting, implicating a highly coordinated, selective EV-mediated delivery mechanism. While small RNA enrichment is clear, determining whether these species are encapsulated within the EV lumen or associated with the vesicle surface will provide critical mechanistic insight through RNase and protease protection assays.

However, it remains to be elucidated in greater detail how the wealth of RXLR and CRN effectors activate or interact with small RNAs in this pathogen and host plant species.

## Conclusion

During early infection, plant multivesicular bodies accumulate in *P. infestans* germ tubes. *Pi-*miR8788 together with a siRNA predicted to target a potato 40S ribosomal gene are selectively enriched in EV fractions, indicating that *P. infestans* exploits host vesicle pathways for targeted small RNA delivery. *StTET8*-enriched plant EVs suppress infection, whereas *CRN*-derived *EDS1*-targeting siRNAs, which were not enriched in EV fractions, employ alternative mechanisms, revealing a sophisticated strategy of pathogenic manipulation of host defenses.

## Data Availability

Publicly available datasets were analyzed in this study. This data can be found here: Raw and processed sequencing data generated in this study were submitted to the NCBI Gene Expression Omnibus (GEO; https://www.ncbi.nlm.nih.gov/geo/) under accession number GSE163382. The smartPARE R package is available online (https://github.com/KristianHoden/smartPARE/), doi:10.5281/zenodo.4495749.

## References

[B1] Amaro T. M. M. M. Thilliez G. J. A. Motion G. B. Huitema E. (2017). A perspective on CRN proteins in the genomics age: evolution, classification, delivery and function revisited. Front. Plant Sci. 8, 99. doi: 10.3389/fpls.2017.00099, PMID: 28217133 PMC5289972

[B2] Åsman A. K. M. Fogelqvist J. Vetukuri R. Dixelius C. (2016). *Phytophthora infestans* Argonaute 1 binds microRNA and small RNAs from effector genes and transposable elements. New Phytol. 211, 993–1007. doi: 10.1111/nph.13946, PMID: 27010746

[B3] Boavida L. C. Qin P. Broz M. Becker J. D. McCormick S. (2013). Arabidopsis tetraspanins are confined to discrete expression domains and cell types in reproductive tissues and form homo- and heterodimers when expressed in yeast. Plant Physiol. 163, 696–712. doi: 10.1104/pp.113.216598, PMID: 23946353 PMC3793051

[B4] Boevink P. C. Birch P. R. J. Turnbull D. Whisson S. C. (2020). Devastating intimacy: the cell biology of plant-*Phytophthora* interactions. New Phytol. 228, 445–458. doi: 10.1111/nph.16650, PMID: 32394464 PMC7540312

[B5] Cai Q. Qiao L. Wang M. He B. Lin F. M. Palmquist J. . (2018). Plants send small RNAs in extracellular vesicles to fungal pathogen to silence virulence genes. Science 360, 1126–1129. doi: 10.1126/science.aar4142, PMID: 29773668 PMC6442475

[B6] Chebli Y. Bidhendi A. J. Kapoor K. Greitmann A. (2021). Cytoskeletal regulation of primary plant cell wall assembly. Curr. Biol. 31, R681–R695. doi: 10.1016/j.cub.2021.03.092, PMID: 34033798

[B7] Corona M. L. Hurbain I. Raposo G. van Niel G. (2023). Characterization of extracellular vesicles by transmission electron microscopy and immunolabeling electron microscopy. Methods Protoc. 2668, 33–43. doi: 10.1007/978-1-0716-3203-1_4, PMID: 37140788

[B8] Couch Y. Buzàs E. I. Di Vizio D. Gho Y. S. Harrison P. Hill A. F. . (2021). A brief history of nearly EV-erything - The rise and rise of extracellular vesicles. J. Extracell. Vesicles 10, e12144. doi: 10.1002/jev2.12144, PMID: 34919343 PMC8681215

[B9] Cui Y. Gao J. He Y. Jiang L. (2020). Plant extracellular vesicles. Protoplasma 257, 3–12. doi: 10.1007/s00709-019-01435-6, PMID: 31468195

[B10] Dagdas Y. F. Pandey P. Tumtas Y. Sanguankiattichai N. Belhaj K. Duggan C. . (2018). Host autophagy machinery is diverted to the pathogen interface to mediate focal defense responses against the Irish potato famine pathogen. eLife 7, e37476. doi: 10.7554/eLife.37476.021, PMID: 29932422 PMC6029844

[B11] de Bary A. (1876). Researches into the nature of the potato fungus Phytophthora infestans. J. R. Soc Agric. England. II 12, 239–269.

[B12] Fabro G. (2022). Oomycete intracellular effectors: specialized weapons targeting strategic plant processes. New Phytol. 233, 1074–1082. doi: 10.1111/nph.17828, PMID: 34705271

[B13] Fry W. (2008). *Phytophthora infestans*: the plant (and *R* gene) destroyer. Mol. Plant Pathol. 9, 385–402. doi: 10.1111/j.1364-3703.2007.00465.x, PMID: 18705878 PMC6640234

[B14] Haas B. J. Kamoun S. Zody M. C. Jiang R. H. Y. Handsaker R. E. Cano L. M. . (2009). Genome sequence and analysis of the Irish potato famine pathogen *Phytophthora infestans*. Nature 461, 393–398. doi: 10.1038/nature08358, PMID: 19741609

[B15] Hu X. Persson Hodén K. Liao Z. Åsman A. Dixelius C. (2022). *Phytophthora infestans* Ago1-associated miRNA promotes potato late blight disease. New Phytol. 233, 443–457. doi: 10.1111/nph.17758, PMID: 34605025

[B16] Huang Y. Wang S. Cai Q. Jin H. (2021). Effective methods for isolation and purification of extracellular vesicles from plants. J. Integr. Plant Biol. 63, 2020–2030. doi: 10.1111/jipb.13181, PMID: 34668639 PMC8972076

[B17] Jahan S. N. Åsman A. K. M. Corcoran P. Fogelqvist J. Vetukuri R. R. Dixelius C. (2015). Plant-mediated gene silencing restricts growth of the potato late blight pathogen *Phytophthora infestans*. J. Exp. Bot. 66, 2785–2794. doi: 10.1093/jxb/erv094, PMID: 25788734 PMC4986879

[B18] Jia J. Lu W. Zhong C. Zhou R. Xu J. Liu W. . (2017). The 25–26 nt small RNAs in *Phytophthora parasitica* are associated with efficient silencing of homologous endogenous genes. Front Microbiol. 8, 773. doi: 10.3389/fmicb.2017.00773, PMID: 28512457 PMC5411455

[B19] Koch B. L. Rutter B. D. Borniego M. L. Singla-Rastogi M. Gardner D. M. Innes R. W. (2025). Arabidopsis produces distinct subpopulations of extracellular vesicles that respond differentially to biotic stress, altering growth and infectivity of a fungal pathogen. J. Extracell. Vesicles 14, e70090. doi: 10.1002/jev2.70090, PMID: 40415221 PMC12104214

[B20] Li X. Y. (2011). Infiltration of *Nicotiana benthamiana* protocol for transient expression via *Agrobacterium*. Bio-Protocol 1, e95. doi: 10.21769/BioProtoc.95, PMID: 37752966

[B21] Li X. Bao H. Wang Z. Wang M. Fan B. Zhum C. . (2018). Biogenesis and function of multivesicular bodies in plant immunity. Front. Plant Sci. 9, 979. doi: 10.3389/fpls.2018.00979, PMID: 30038635 PMC6047128

[B22] Liu T. Ye W. Ru Y. Yang X. Gu B. Tao K. . (2011). Two host cytoplasmic effectors are required for pathogenesis of *Phytophthora sojae* by suppression of host defenses. Plant Physiol. 155, 490–501. doi: 10.1104/pp.110.166470, PMID: 21071601 PMC3075790

[B23] More K. Klinger C. M. Barlow L. D. Dacks J. B. (2020). Evolution and natural history of membrane trafficking in eukaryotes. Curr. Biol. 30, R553–R564. doi: 10.1016/j.cub.2020.03.068, PMID: 32428497

[B24] Nowicki M. Foolad M. R. Nowakowska M. Kozik E. U. (2012). Potato and tomato late blight caused by *Phytophthora infestans*: An overview of pathology and resistance breeding. Plant Dis. 96, 4–17. doi: 10.1094/PDIS-05-11-0458, PMID: 30731850

[B25] Otsuga D. Keegan B. R. Brisch E. Thatcher J. W. Hermann G. J. Bleazard W. . (1998). The dynamin-related GTPase, Dnm1p, controls mitochondrial morphology in yeast. J. Cell Biol. 143, 333–349. doi: 10.1083/jcb.143.2.333, PMID: 9786946 PMC2132834

[B26] Pais M. Yoshida K. Giannakopoulou A. Pel M. A. Cano L. M. Oliva R. F. . (2018). Gene expression polymorphism underpins evasion of host immunity in an asexual lineage of the Irish potato famine pathogen. BMC Ecol. Evol. 18, 93. doi: 10.1186/s12862-018-1201-6, PMID: 29973156 PMC6032779

[B27] Persson Hodén K. Hu X. Martinez G. Dixelius C. (2021). smartPARE: an R package for efficient identification of true mRNA cleavage sites. Int. J. Mol. Sci. 28, 4267. doi: 10.3390/ijms22084267, PMID: 33924042 PMC8073297

[B28] Qin S. Li W. Zeng J. Huang Y. Cai Q. (2024). Rice tetraspanins express in specific domains of diverse tissues and regulate plant architecture and root growth. Plant J. 117, 892–908. doi: 10.1111/tpj.16536, PMID: 37955978

[B29] Rubiato H. M. Liu M. O’Connell R. J. Nielsen M. E. (2022). Plant SYP12 syntaxins mediate an evolutionarily conserved general immunity to filamentous pathogens. eLife 11, e73487. doi: 10.7554/eLife.73487, PMID: 35119361 PMC8865848

[B30] Rutter B. D. Innes R. W. (2017). Extracellular vesicles isolated from the leaf apoplast carry stress-response proteins. Plant Physiol. 173, 728–741. doi: 10.1104/pp.16.01253, PMID: 27837092 PMC5210723

[B31] Schlacht A. Herman E. K. Klute M. J. Filed M. C. Dacks J. B. (2014). Missing pieces of an ancient puzzle: evolution of the eukaryotic membrane-trafficking system. Cold Spring Harbor Persp. Biol. 6, a016048. doi: 10.1101/cshperspect.a016048, PMID: 25274701 PMC4176009

[B32] Singh S. Hu X. Dixelius C. (2023). Dynamics of nucleic acid mobility. Genetics 225, iyad132. doi: 10.1093/genetics/iyad132, PMID: 37491977 PMC10471207

[B33] Stotz H. U. Brotherton D. Inal J. (2022). Communication is key: extracellular vesicles as mediators of infection and defence during host–microbe interactions in animals and plants 46, fuab044. doi: 10.1093/femsre/fuab044, PMID: 34448857 PMC8767456

[B34] Torto T. A. Li S. Styer A. Huitema E. Testa A. Gow N. A. . (2003). EST mining and functional expression assays identify extracellular effector proteins from the plant pathogen *Phytophthora*. Genome Res. 13, 1675–1685. doi: 10.1101/gr.910003, PMID: 12840044 PMC403741

[B35] Ueda T. Yamaguchi M. Uchimiya H. Nakano A. (2001). Ara6, a plant-unique novel type Rab GTPase, functions in the endocytic pathway of *Arabidopsis thaliana*. EMBO J. 20, 4730–4741. doi: 10.1093/emboj/20.17.4730, PMID: 11532937 PMC125591

[B36] Van Niel G. Raposo G. (2018). Shedding light on the cell biology of extracellular vesicles. Nat. Rev. Mol. Cell Biol. 19, 213–228. doi: 10.1038/nrm.2017.125, PMID: 29339798

[B37] Vetukuri R. R. Avrova A. O. Grenville-Briggs L. J. Van West P. Söderbom F. Savenkov E. I. . (2011). Evidence for involvement of Dicer-like, Argonaute and histone deacetylase proteins in gene silencing in *Phytophthora infestans*. Mol. Plant Pathol. 12, 772–785. doi: 10.1111/j.1364-3703.2011.00710.x, PMID: 21726377 PMC6640358

[B38] Wang S. Boevink P. C. Welsh L. Zhang R. Whisson S. C. Birch P. R. J. (2017). Delivery of cytoplasmic and apoplastic effectors from *Phytophthora infestans* haustoria by distinct secretion pathways. New Phytol. 216, 205–215. doi: 10.1111/nph.14696, PMID: 28758684 PMC5601276

[B39] Wang S. McLellan H. Bukharova T. He Q. Murphy F. Shi J. . (2019). Phytophthora infestans RXLR effectors act in concert at diverse subcellular localisations to enhance host colonisation. J. Exp. Bot. 70, 343–356. doi: 10.1093/jxb/ery360, PMID: 30329083 PMC6305197

[B40] Wang F. Shang Y. Fan B. Yu J.-Q. Chen Z. (2014). Arabidopsis LIP5, a positive regulator of multivesicular body biogenesis, is a critical target of pathogen-responsive MAPK cascade in plant basal defense. PloS Pathog. 10, e1004243. doi: 10.1371/journal.ppat.1004243, PMID: 25010425 PMC4092137

[B41] Wang S. Welsh L. Thorpe P. Whisson S. C. Boevink P. C. Birch P. R. J. (2018). The *Phytophthora infestans* haustorium is a site for secretion of diverse classes of infection-associated proteins. mBio 9, e01216–e01218. doi: 10.1128/mBio.01216-18, PMID: 30154258 PMC6113627

[B42] Wang Z. Zeng J. Deng J. Hou X. Zhang J. Yan W. . (2023). Pathogen-derived extracellular vesicles: emerging mediators of plant-microbe interactions. Mol. Plant-Microbe Interact. 36, 218–227. doi: 10.1094/MPMI-08-22-0162-FI, PMID: 36574017

[B43] Whisson S. C. Boevink P. C. Wang S. Birch P. R. J. (2016). The cell biology of late blight disease. Curr. Opin. Microbiol. 34, 127–135. doi: 10.1016/j.mib.2016.09.002, PMID: 27723513 PMC5340842

[B44] Yuan G. Gao H. Yang T. (2023). Exploring the role of the plant actin cytoskeleton: From signaling to cellular functions. Int. J. Mol. Sci. 24, 15480. doi: 10.3390/ijms242015480, PMID: 37895158 PMC10607326

[B45] Yuen E. L. H. Shepherd S. Bozkurt T. O. (2023). Traffic control: Subversion of plant membrane trafficking by pathogens. Ann. Rev. Phytopathol. 61, 325–350. doi: 10.1146/annurev-phyto-021622-123232, PMID: 37186899

[B46] Zerial M. McBride. H. (2001). Rab proteins as membrane organizers. Nat. Rev. Mol. Cell Biol. 2, 107–117. doi: 10.1038/35052055, PMID: 11252952

[B47] Zhang D. Burroughs A. M. Vidal N. D. Iyer L. M. Aravind L. (2016). Transposons to toxins: the provenance, architecture and diversification of a widespread class of eukaryotic effectors. Nucleic Acids Res. 44, 3513–3533. doi: 10.1093/nar/gkw221, PMID: 27060143 PMC4857004

