# Supplementary material for: A Phytophthora infestans CRN1-derived small RNA is predicted to target the potato immune regulator EDS1
Source: Front Plant Sci. 2026 Apr 10;17:1791978. doi: 10.3389/fpls.2026.1791978 (PMC13106546; doi:10.3389/fpls.2026.1791978)
Supplement: Supplementary file 1 [file DataSheet1.pdf]

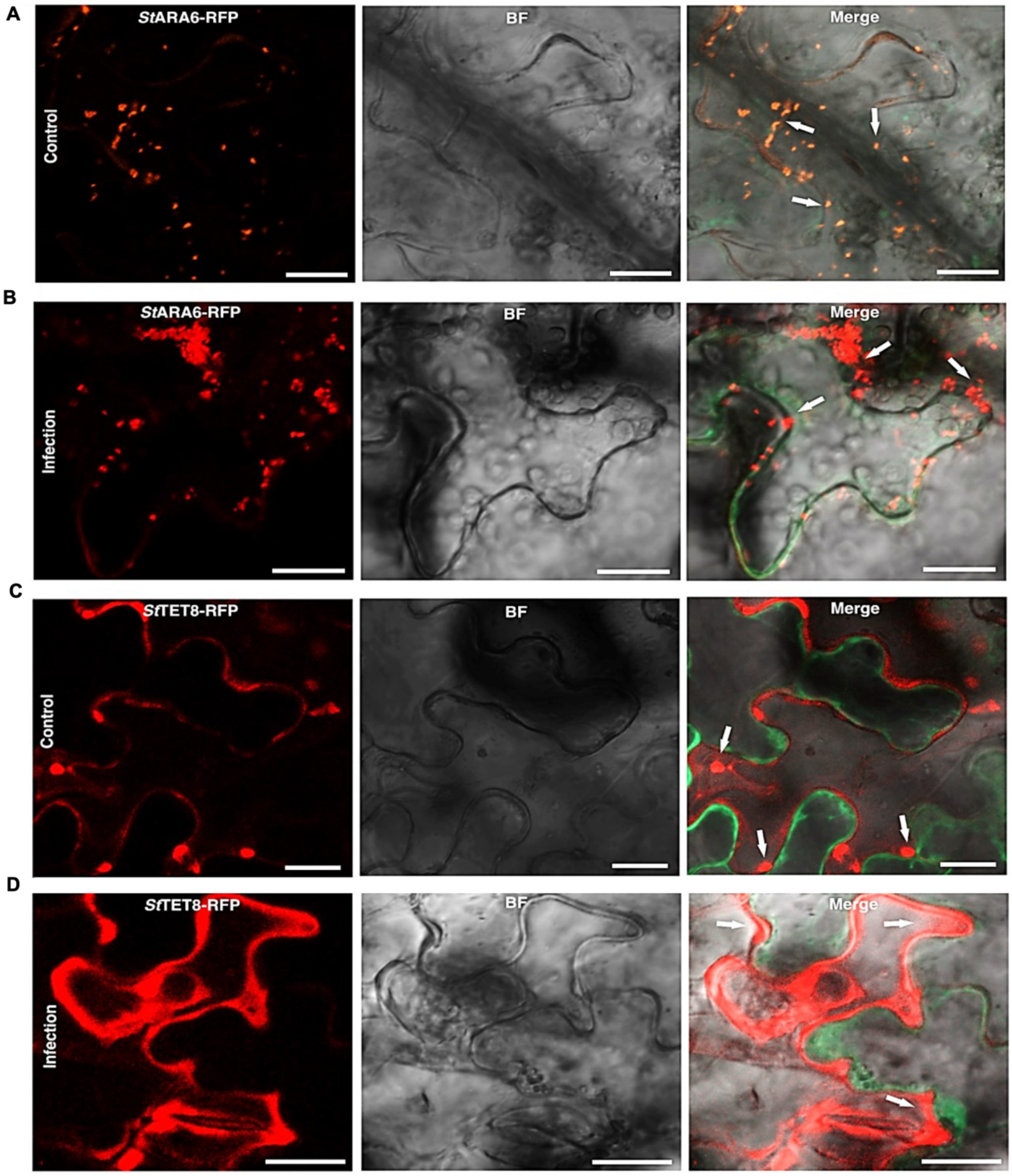

Figure S1. Accumulation of extracellular vesicles (EVs) in response to *Phytophthora infestans* infection. Agroinfiltration of *Nicotiana benthamiana* leaves using plasmids expressing StARA6-RFP and StTET8-RFP markers. (A) StARA6-RFP localization in uninfected control leaves, and (B) at 5.0 days post-infection (dpi). (C) StTET8-RFP localization in uninfected control leaves, and (D) at 5.0 dpi. Images were captured at 5.0 dpi using a confocal microscope (Leica SP8). Scale bar represents 20 µm. White arrows indicate EV accumulation sites. Representative images are shown from a minimum of four independent biological replicates, with consistent observations across multiple focal planes per replicate.

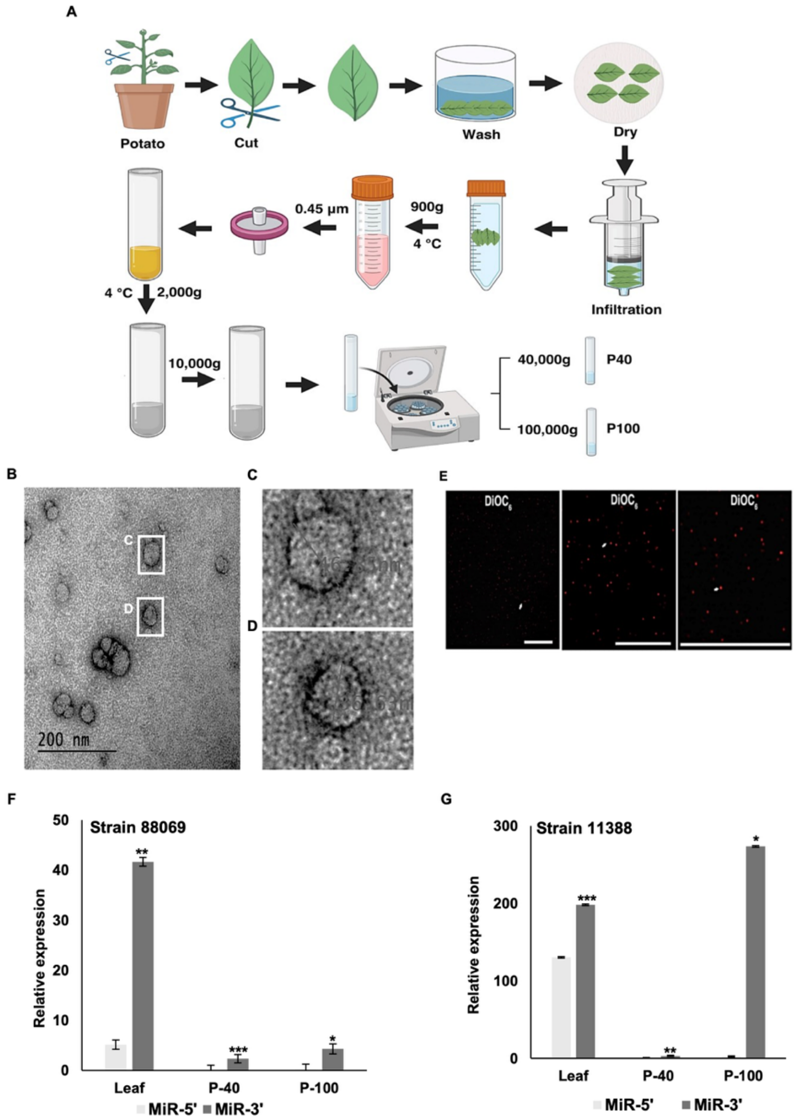

Figure S2. Extracellular vesicles (EVs) from infected potato leaves and their characterization. (A) Schematic of the stepwise centrifugation protocol for EV isolation. Young potato leaves were sampled at 5 days post-infection (dpi), rinsed, and dried. Apoplastic fluid was collected by centrifugation at 500 × *g* for 10 min (to remove debris and broken cells), followed by 12,000 × *g* for 20 min (to remove large cells and organelles), then 100,000 × *g* (P100 pellet, containing EVs) and 40,000 × *g* (P40 pellet) for 90 min each. The entire procedure requires approximately 2 days. Illustration created using bioRender.com. (B) Transmission electron microscopy (TEM) of EVs from different centrifugation fractions shown in (A). Scale bar represents 200 nm. (C, D) Enlarged views of individual EVs indicated by white boxes in panel (B). Measured diameters are 46.8 nm (C) and 36.6 nm (D), representative of the 36.6–46.8 nm size range observed across all samples. (E) EV samples stained with DiOC6 (3,3′-dihexyloxacarbocyanine iodide) and visualized by confocal microscopy at different magnifications. The arrow indicates EVs stained with the membrane-specific probe. Scale bar represents 50 μm. Representative images from three independent biological replicates are shown. (F, G) Relative expression levels of Pi-miR8788 measured by quantitative reverse transcription-polymerase chain reaction (qRT-PCR) in EV-enriched fractions (P40 and P100 pellets) and total leaf extracts from potato leaves infected with *P. infestans* strain 88069 (F) or 11388 (G). Light gray bars represent the miR-5′ strand, dark gray bars represent the miR-3′ strand. Total leaf extracts represent the complete RNA profile of whole tissue before EV purification, whereas P40 and P100 fractions contain RNA specifically associated with isolated vesicles. Data represent mean ± standard deviation from three independent biological replicates (n = 3), each with three technical replicates. Expression levels were normalized to the housekeeping gene *StActin*. Statistical analysis was performed using Student's *t*-test comparing EV fractions to total leaf extracts: $P < 0.05$; *$P < 0.01$; **$P < 0.001$.

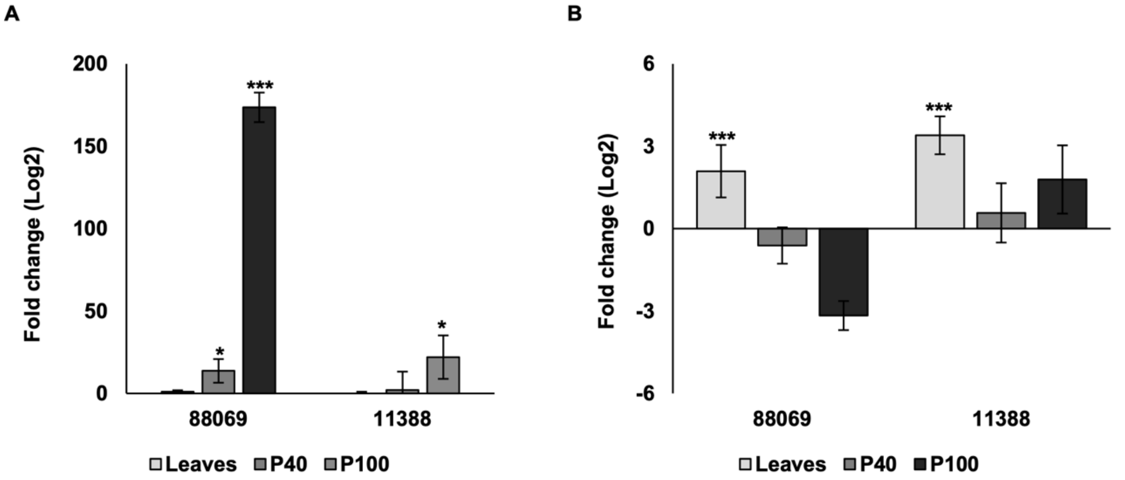

Figure S3. Relative expression levels of *P. infestans*-derived small RNAs in leaf tissue and extracellular vesicle (EV)-enriched fractions. Relative expression levels of small RNAs in total leaf samples (leaves) and extracellular vesicle (EV)-enriched fractions (P40 and P100 pellets) from potatoes infected with *P. infestans*. (A) Pi-siRNA 5′-AACTACTCCATGAATGTCTCC-3′, a *P. infestans*-derived small RNA predicted to target the potato 40S ribosomal protein S8 gene (PGSC0003DMT400053172). This siRNA was selectively enriched in the P100 fraction, indicating association with EVs. (B) *Pi*-siRNA 5′-TTGGAGAGATGGAAAGACGG-3′, derived from the *P. infestans* CRN effector PITG_04767, predicted to target the potato *EDS1* gene (PGSC0003DMT400083030; enhanced disease susceptibility 1). This *CRN*-derived siRNA was detected primarily in total leaf extracts and not enriched in EV fractions. Data represent mean ± standard deviation (n = 3 biological replicates). Expression was normalized to that of *StActin*. Statistical analysis was performed using Student's *t*-test: *P* < 0.05; **P* < 0.01; ***P* < 0.001.

Table S1. List of cloning oligos used in different assays in this study.

Oligo Name	Sequence (5′ → 3′)
**Cloning primers - Gateway**	
StAGO1bTGAGW_F	GGGGACAAGTTTGTACAAAAAAGCAGGCTTCATGGTGAGGAAGAGGAGAACTG
StAGO1bTGAGW_R	GGGGACCACTTTGTACAAGAAAGCTGGGTCCTAGCAATAGAACATGACCCTCT
StAGO1bNOTGAGW_R	GGGGACCACTTTGTACAAGAAAGCTGGGTCGCAATAGAACATGACCCTCTTCA
StAGO1aTGAGW_F	GGGGACAAGTTTGTACAAAAAAGCAGGCTTCATGGTGCGGAAGAGGAGAAC
StAGO1aTGAGW_R	GGGGACCACTTTGTACAAGAAAGCTGGGTCCTAACAATAGAACATCACCCTTTTGAC
StAGO1aNOTGAGW_R	GGGGACCACTTTGTACAAGAAAGCTGGGTCACAATAGAACATCACCCTTTTGACA
PinAGO1allGW_F	GGGGACAAGTTTGTACAAAAAAGCAGGCTTCATGCCGGGGCGGCGAAACAAGCAGCAAGACT C
PinAGO1allGW_R	GGGGACCACTTTGTACAAGAAAGCTGGGTCTTACACGAAGTACATCTCGCTCTT
T8TTGAallGW_F	GGGGACAAGTTTGTACAAAAAAGCAGGCTTCATGGTGCGTTGTAGCAACAATTTAGTGGGGA
T8TTGAallGW_R	GGGGACCACTTTGTACAAGAAAGCTGGGTCTTAAGGATAACGCTTCCAAGCATTGTCCTCTC
T8TNOTGAallGW_R	GGGGACCACTTTGTACAAGAAAGCTGGGTCAGGATAACGCTTCCAAGCATTGTCCTCTCGG
Rab5TGAallGW_F	GGGGACAAGTTTGTACAAAAAAGCAGGCTTCATGGGTTGCGCATCTTCAGC
Rab5TGAallGW_R	GGGGACCACTTTGTACAAGAAAGCTGGGTCTCAAGCAGCAGTCGGGCGTGGCAATC
Rab5NOTGAallGW_R	GGGGACCACTTTGTACAAGAAAGCTGGGTCAGCAGCAGTCGGGCGTGGCAATC
CRN1TGAGW_F	GGGGACAAGTTTGTACAAAAAAGCAGGCTTCATGGTGGCGCGGAAATGGTTC
CRN1TGAGW_R	GGGGACCACTTTGTACAAGAAAGCTGGGTCCTAACGCGAATCCGTGTTGAATTTGAA
CRN1NOTGAGW_R	GGGGACCACTTTGTACAAGAAAGCTGGGTCACGCGAATCCGTGTTGAATTTGAA
CRN2TGAGW_F	GGGGACAAGTTTGTACAAAAAAGCAGGCTTCATGGCGGCGCGGAAATGGTTC
CRN2TGAGW_R	GGGGACCACTTTGTACAAGAAAGCTGGGTCCTAACGCGAATCCGTGTTGAATTTGAA
CRN2NOTGAGW_R	GGGGACCACTTTGTACAAGAAAGCTGGGTCACGCGAATCCGTGTTGAATTTGAA
CRN3TGAGW_F	GGGGACAAGTTTGTACAAAAAAGCAGGCTTCATGGTGAAATTTTTCTGTGCT
CRN3TGAGW_R	GGGGACCACTTTGTACAAGAAAGCTGGGTCTCACGTGGATTCACTTCCACTGGCG
CRN3NOTGAGW_R	GGGGACCACTTTGTACAAGAAAGCTGGGTCACGTGGATTCACTTCCACTGGCG
**qPCR primers - reference genes**	
StACT101_F	GCCTCCTGAACGGAAGTACA
StACT101_R	AATGGAAGGACCGGATTCAT

**Oligo Name**	**Sequence (5′ → 3′)**
**Stem-loop RT primers - small RNAs**	
miR8788-5'	GTCGTATCCAGTGCAGGGTCCGAGGTATTCGCACTGGATACGACCGGCGC
miR8788-3'	GTCGTATCCAGTGCAGGGTCCGAGGTATTCGCACTGGATACGACGCGTAC
Pin_sRNA (Pi-siRNA targeting 40S RPS8)	GTCGTATCCAGTGCAGGGTCCGAGGTATTCGCACTGGATACGACTTGATG
Pin_sRNA_143 (CRN1-derived siRNA targeting EDS1	GTCGTATCCAGTGCAGGGTCCGAGGTATTCGCACTGGATACGACAACCTC
**qPCR primers- small RNAs**	
pin-5'_F	TCGCGCTACCAAGCGTACCA
pin-3'_R	TTCCTGCGCTGGTTCGCTTG
Pin_sRNA_F (Pi-siRNA targeting 40S RPS8)	GGTGCAACTACTCCATGAATGTCTCC
Pin_sRNA_143_F (CRN1-derived siRNA targeting EDS1)	GGTGCTTGGAGAGATGGAAAGACG
Universal Reverse Primer	GTGCAGGGTCCGAGGT

Table S2. *P. infestans* siRNAs predicted to target potato genes.

***P. infestans* siRNAs**	**Targeted gene**	**Targeted gene function**	**NSRA**	**NSRA_C**
AACTACTCCATGAATGTCTCC*	PGSC0003DMT400053172	40S ribosomal protein S8	25.8	25.8
TACATCATCCTCGTTAGCTAG	PGSC0003DMT400060590	Transducin family protein	23	0
AGGTGAGCTCAATGAGAACA	PGSC0003DMT400032491	L-lactate dehydrogenase	19.65	0
GGGTATTCATATTTCAGCG	PGSC0003DMT400012820	Conserved gene of unknown function	16	0
TGGATGAAGCAGAGTCTGGT	PGSC0003DMT400029517	Transport inhibitor response 1	13	0
TTGGAGAGATGGAAAGACGG	PGSC0003DMT400083030	Enhanced disease susceptibility 1 protein	12.5	0
CAGCATAATCGTATTGGACTT	PGSC0003DMT400051686	Step II splicing factor slu7	9.67	0
TACTTCCCGTTCCACGTGTGC	PGSC0003DMT400018765	Isoamylase isoform 3	9.4	0
AGCAAGGTTGATCTCGGCGC	PGSC0003DMT400037240	Dimethylallyltransferase	8.53	0
TGTGCAGCTGTTTCTCATCG	PGSC0003DMT400033370	Conserved gene of unknown function	7.9	0
GAGTTTGAGCACATATGTTGG	PGSC0003DMT400071268	Eukaryotic translation initiation factor	7.9	0
TGAAGGCAGCTTGTAAGAACT	PGSC0003DMT400013692	Alpha-glucosidase	3.75	0
CGCCGCCTCCTCCATGGGCAC	PGSC0003DMT400004028	Glycine-rich protein 2	3.65	0
CACATTGCTGGACAATTCTCT	PGSC0003DMT400041692	LRR receptor-like serine/threonine-protein kinase	3.65	0
CTCTTGTTGTGCCGGAGAGA	PGSC0003DMT400045157	ARF GTPase-activating protein	3.65	0
ATCATCTTCTTAGCTTTCTCT	PGSC0003DMT400062863	Ubiquitin-protein ligase	3.6	0
TTGGAGAGATGGAAAGACGG A	PGSC0003DMT400083030	Enhanced disease susceptibility 1 protein	3.4	0
TGGCTGCTGAAGGCTGTGTT	PGSC0003DMT400048410	F-box/LRR-repeat protein 15	3.3	0
ACTTTAACATGCTCAATTGCT	PGSC0003DMT400055157	Phytosulfokine peptide	3.2	0
TCCATCCATGTTGGTCGCTGG	PGSC0003DMT400067668	Ring finger protein	3.1	0
AGCTTTTGCATTGCCTGCTCT	PGSC0003DMT400052108	Bromodomain-containing protein	3	0
CTGTGTTGGTGTTTATGCGT	PGSC0003DMT400054926	Conserved gene of unknown function	2.85	0
TGAGTTTGAGCACATATGTTG	PGSC0003DMT400071268	Eukaryotic translation initiation factor	2.75	0

AACAAATGGGTGAGAGGCTGT	PGSC0003DMT400010350	Splicing factor, arginine/serine-rich	2.6	0
CATACATTTCAATATCGTGGT	PGSC0003DMT400084781	Anthranilate N-benzoyltransferase protein	2.6	0
GTCCGTCTTTCCATCTCTCC	PGSC0003DMT400092196	Elongation factor TuB, chloroplastic	2.6	0
TGGATGAAGCAGAGTCTGG	PGSC0003DMT400029517	TIR1	2.45	0
TGGCTGCTGAAGGCTGTGT	PGSC0003DMT400048411	F-box/LRR-repeat protein 15	2.45	0
TTGGAGAGATGGAAAGACG	PGSC0003DMT400083030	Enhanced disease susceptibility 1 protein	2.45	0
ATCACCAGCTCTCTACTGTC	PGSC0003DMT400010886	Thaumatin	2.3	0
TGACTGGAGGGGAGAATGG	PGSC0003DMT400024848	Ice binding protein	2.2	0
TCAAAGTCAAACCAGTTACAG	PGSC0003DMT400034920	Conserved gene of unknown function	2.2	0
TCCATCCATGTTGGTCGCTG	PGSC0003DMT400067668	Ring finger protein	2.2	0
TTTGACGACTTGCAAGCGCTG	PGSC0003DMT400078451	Wall-associated kinase	2.2	0
AAAATCCTGGCTAGAGATTTA	PGSC0003DMT400079210	Serine/threonine-protein kinase SAPK1	2.1	0
AAAATCCTGGCTAGAGATTTA	PGSC0003DMT400079211	Serine/threonine-protein kinase SAPK1	2.1	0
CTACATCAACGCTCTGTCGG	PGSC0003DMT400008203	NERD domain containing protein	1.9	0
TGTCTTGTGCGCTTGTCTTG	PGSC0003DMT400032713	Conserved gene of unknown function	1.9	0
AGCAAGGTTGATCTCGGCGCT	PGSC0003DMT400037240	Dimethylallyltransferase	1.9	0
CTGTGTTGGTGTTTATACGTTG	PGSC0003DMT400054925	Conserved gene of unknown function	1.9	0
CGCGAGACGGGGGTGAGGT	PGSC0003DMT400059397	Protein SIS1	1.9	0
TGCAGCACCATCTTGTTCTTT	PGSC0003DMT400083856	Conserved gene of unknown function	1.9	0

*siRNA used in qRT-PCR.

NSRA indicates the normalized small RNA abundance.

NSRA_C indicates the normalized small RNA abundance in the control.

Table S3. *P. infestans* effector siRNAs predicted to target potato genes.

P. infestans **siRNA**	**Source gene**	**Effector type**	**Potato gene predicted to be siRNA target**	**Potato gene function**	**NSRA**	**NSRA_C**
TTGGAGAGATGGAAAGACGG*	PITG_04767	CRN1	PGSC0003DMT400083030	Enhanced disease susceptibility 1 protein	12.5	0
TTATGTTGGATCATGCGAAT	PITG_04769	CRN2	PGSC0003DMT400076806	Protease degS	2.2	2.2
TGCAGCACCATCTTGTTCTTT	PITG_09043	CRN3**	PGSC0003DMT400083856	Conserved gene of unknown function	1.9	0
AACGACTGGTGCGAATTGG	PITG_04743	pseudo-CRN	PGSC0003DMT400029178	HDDC2 protein	2.7	0.21
GTCCGTCTTTCCATCTCTCC	PITG_04736	pseudo-CRN	PGSC0003DMT400092196	Elongation factor TuB, chloroplastic	0	0.28
CAGCTTGTGGGGAGTGTGGC	PITG_01398	ENZYME HYDROLASE	PGSC0003DMT400083469	Bacterial spot disease resistance protein 4	3.2	0.7

*siRNA used in qRT-PCR

**CRN3 encodes the LFLAK domain.

NSRA indicates the normalized small RNA abundance.

NSRA_C indicates the normalized small RNA abundance in the control.
